# Perceived socioeconomic vulnerability, but not objective poverty, is linked to interoception through perceived stress

**DOI:** 10.3389/fpsyg.2026.1713385

**Published:** 2026-04-01

**Authors:** Daniel Franco-O’Byrne, Jorge Eduardo Ferdman, David Huepe

**Affiliations:** 1 Latin American Brain Health Institute (BrainLat), Universidad Adolfo Ibáñez, Santiago, Chile; 2 Center for Social and Cognitive Neuroscience (CSCN), School of Psychology, Universidad Adolfo Ibáñez, Santiago, Chile; 3 School of Psychology, Universidad Adolfo Ibáñez, Santiago, Chile

**Keywords:** allostatic interoception, interoception, perceived socioeconomic status, socioeconomic status, stress

## Abstract

**Introduction:**

Socioeconomic vulnerability is associated with higher levels of stress and adverse effects on physical, mental, and cognitive health. However, its influence on interoceptive awareness—defined as the perception, interpretation, and regulation of bodily signals—remains underexplored. This study examined the relationships between objective (multidimensional poverty) and subjective (perceived vulnerability) measures of socioeconomic vulnerability, perceived stress, and interoceptive awareness, as well as the mediating role of stress.

**Methods:**

A total of 104 adults (50 women, 54 men; aged 30–45 years; mean schooling = 14.7 years) completed self-report measures of perceived vulnerability, perceived stress, and interoceptive awareness using the Multidimensional Assessment of Interoceptive Awareness (MAIA).

**Results:**

Perceived vulnerability, but not multidimensional poverty, was negatively associated with interoceptive awareness, both at the total MAIA score level and across subscales. Furthermore, perceived stress partially mediated the association between perceived vulnerability and interoceptive awareness.

**Discussion:**

These findings suggest that the subjective perception of socioeconomic vulnerability may impair the ability to attend to and consciously use bodily signals through psycho-affective and cognitive mechanisms. This complements physiological models linking socioeconomic experiences with interoceptive processes, highlighting the relevance of subjective vulnerability in shaping interoceptive functioning.

## 1 Introduction

Socioeconomic vulnerability, understood as disadvantage in income, resources, opportunities, and access to services (Hasan et al., 2024; Palermos et al., 2024; Park and Ko, 2021; Srivastava and Muhammad, 2022), is associated with higher levels of stress and negative consequences for physical, mental, and cognitive health (Hoebel and Lampert, 2020; Navarro-Carrillo et al., 2020; Präg et al., 2016). While objective indicators of socioeconomic status have been documented to relate to these outcomes (Barradas et al., 2021; Kivimäki et al., 2020), a growing number of studies highlight that subjective measures—such as perceived socioeconomic vulnerability (PV) or perceived social status—more consistently predict well-being, health, and cognition (Gruenewald et al., 2006; Kim et al., 2021; Muhammad et al., 2022; Peretz-Lange et al., 2022; Ursache et al., 2015), even when controlling for objective indicators (Quon and McGrath, 2014; Zhao et al., 2023). Various studies show that objective socioeconomic status is linked to reductions in interoception—conceived as the ability to perceive, interpret, and use internal bodily signals for self-regulation (e.g., heart rate, breathing, hunger; Alhadeff and Yapici, 2024; Paulus et al., 2019)—both at the level of physiological sensitivity (Alvarez et al., 2022; Leão et al., 2025; Santamaría-García et al., 2024) and bodily signal perception (Chentsova-Dutton and Dzokoto, 2014). Nevertheless, the literature has focused primarily on non-conscious physiological markers, leaving conscious interoception (e.g., attention to, awareness of, and confidence in bodily signals) insufficiently characterized. Consequently, although the relevance of subjective measures of socioeconomic vulnerability is recognized, it remains unknown to what extent perceived socioeconomic vulnerability (PV)—beyond objective indicators—is associated with conscious interoception, and whether its effect differs from that observed for objective socioeconomic status.

Interoception manifests consciously through skills of interoceptive accuracy, sensitivity, and awareness, but it also encompasses unconscious physiological processes that sustain homeostasis (Mehling et al., 2013; Berntson and Khalsa, 2021). Interoceptive awareness facilitates the attention to and processing of bodily signals, contributing to affect regulation, decision-making, and the integration of emotional states (Mehling et al., 2012; Fiskum et al., 2023; Verdejo-Garcia et al., 2012), while also functioning as a bridge between the internal and external worlds (Barrett and Simmons, 2015; Quigley et al., 2020)

The allostatic–interoceptive model (Migeot et al., 2023; Santamaría-García et al., 2024 (Franco-O’Byrne et al., 2024) provides a theoretical framework for understanding how socioeconomic vulnerability may affect interoception. This model suggests that exposure to socioeconomically vulnerable contexts dysregulates allostatic–interoceptive loops, compromising both ascending interoceptive signals and top-down central control (Alvarez et al., 2022; Schulz and Vögele, 2015; Santamaría-García et al., 2024). However, these models rarely integrate the conscious dimension of interoception, focusing mainly on physiological indicators. This gap underscores the need to explore how the subjective perception of socioeconomic vulnerability may impact conscious interoception.

Given that PV is associated with higher levels of perceived stress (Kim et al., 2021) and considering that stress constitutes a central mechanism in the allostatic–interoceptive model, it can be posited as a plausible mediator of the relationship between PV and interoceptive awareness. Nevertheless, the question remains whether the effects on stress and, potentially, on interoception differ depending on whether vulnerability is assessed through structural SES indicators (e.g., income, education) or through the subjective perception of vulnerability. While objective measures capture material conditions and available resources (Navarro-Carrillo et al., 2020; Rakesh et al., 2024; Zhao et al., 2023), PV incorporates cognitive evaluations, social comparisons, and psychosocial experiences that could amplify the stress response (Hoebel and Lampert, 2020; Hooker et al., 2017; Nobles et al., 2013; Steen et al., 2020). This distinction allows for the anticipation of specific pathways through which PV may affect conscious interoception, beyond those mediated by stress, opening the possibility of differential effects of objective and subjective measures on interoceptive processes.

In this context, the present study aims to understand how objective measures (multidimensional poverty) and subjective measures (perceived socioeconomic vulnerability) relate to conscious interoception, as well as to evaluate the role of perceived stress as a possible mediator of these relationships. Based on the previously discussed evidence showing that subjective evaluations of vulnerability capture psychosocial and emotional aspects not reflected by objective measures (Kim et al., 2021; Kraft and Kraft, 2023; Steen et al., 2020), as well as the central role of stress in the impact of socioeconomic experiences on interoceptive processes (Alvarez et al., 2022; Leão et al., 2025), it is proposed that perceived vulnerability is more strongly associated with conscious interoception than multidimensional poverty and that perceived stress mediates this relationship. These findings complement current physiological models linking socioeconomic influences with interoceptive abilities, contributing to an understanding of the psycho-affective and cognitive mechanisms associated with interoception in socioeconomically vulnerable populations.

## 2 Materials and methods

### 2.1 Research design

This study employed a cross-sectional correlational design to examine the associations among perceived vulnerability, perceived stress, and interoception.

### 2.2 Participants

The sample consisted of 104 participants (50 women and 54 men), representative of the general Chilean population between 30 and 45 years of age. Years of education (defined as total completed years of formal schooling) averaged 14.7 (SD = 3.43; median = 14, IQR = 12–17; range = 4–23). All participants provided written informed consent prior to participation, and the study was approved by the institutional ethics committee, in accordance with the guidelines of the Declaration of Helsinki for research involving human subjects.

As exclusion criteria, individuals with visual and/or hearing impairments that would prevent them from completing the various tasks and measurements of the study, as well as those with psychiatric or neurological histories that could interfere with the evaluation of the protocol, were considered.

### 2.3 Instruments

#### 2.3.1 Stress

The Perceived Stress Scale (PSS) (Cohen et al., 1983), in its Spanish version (Remor, 2006), was used to measure the extent to which individuals appraise situations in their lives as stressful. These situations are divided into three aspects considered central components of the stress experience, namely, the degree to which people perceive life as unpredictable, uncontrollable, or overloaded (Remor, 2006). The scale consists of 14 items with a 5-point Likert-type response format ranging from 0 (never) to 4 (very often), and it demonstrated adequate reliability in our sample (Cronbach’s α = 0.719).

#### 2.3.2 Interoception

Regarding interoception, the Spanish version of the Multidimensional Assessment of Interoceptive Awareness (MAIA) (Mehling et al., 2012) was used. This multidimensional instrument consists of 32 items evaluated on a Likert-type scale with six ordinal response levels coded from 0 (never) to 5 (always), except for items 5, 6, 7, 8, and 9, which are reverse-scored, followed by the calculation of a total score for each participant (Valenzuela-Moguillansky and Reyes-Reyes, 2015). In the present study, the instrument demonstrated good reliability (Cronbach’s α = 0.733).

#### 2.3.3 Perceived and objective socioeconomic status

To measure perceived and objective socioeconomic status, the perceived vulnerability (PV) and multidimensional poverty (MP) subscales were extracted from the Social Determinants of Health questionnaire (Piña-Escudero et al., 2023).

The Multidimensional Poverty dimension consists of four subscales: “Limitations to Basic Needs,” “Monthly economic stability,” “Health Access Deprivation,” “Food Insecurity,” and “Quality of nutrition.” Each subscale included 3 items that assessed difficulties related to economic constraints and access to goods or essential services across three life stages (0–10 years, 35–45 years, and the last year). Items were rated on a 0–2 scale (0 = “not difficult at all,” 1 = “somewhat difficult,” 2 = “very difficult”), allowing each subscale to yield a maximum score of 6 points, with higher scores indicating greater deprivation within that domain. The total scale score ranged from 0 to 30, reflecting cumulative multidimensional socioeconomic difficulties. Internal consistency was good, with a Cronbach’s α = 0.813.

The Perceived Vulnerability (PV) dimension consisted of four items assessing participants’ perceived social standing relative to others in their community across different stages of the life course. Responses were provided on a scale from 1 to 10. In the original scale, lower values indicated a lower perceived position; however, for the purposes of this study, items were reverse-scored so that higher scores reflected greater perceived vulnerability. This subscale showed good internal consistency in our sample (Cronbach’s α = 0.815).

### 2.4 Procedure

Participants were initially contacted via telephone and flyers distributed in community settings. Eligibility was pre-screened through an enrolment link and/or telephone interview to ensure compliance with inclusion criteria. Eligible participants were invited to attend an in-person laboratory session.

Upon arrival, participants were seated in a quiet room equipped with a desk and computer to minimize environmental distractions during data collection. A trained psychologist administered the study instruments and supervised the completion of the self-report questionnaires. Data was recorded directly into a secure digital system. The questionnaires were administered in a fixed order and completed in a single session.

The full protocol, including screening confirmation and questionnaire completion, required approximately 90 min on average. Participants received compensation upon completion of the session.

The study protocol was approved by the Universidad Adolfo Ibañez Ethics Committee (No. 26/2023). All participants provided written informed consent prior to participation.

## 3 Data analysis

Descriptive statistics (means, standard deviations, and ranges) were calculated for all study variables. Normality assumptions were assessed using the Shapiro–Wilk test. Given deviations from normality, nonparametric analyses were conducted. Spearman’s rank-order correlations were computed to examine bivariate associations among variables.

Mediation analysis was conducted using a rank-based nonparametric approach with bootstrapping to estimate indirect effects via a product-of-coefficients framework. Further methodological details are provided in the following subsection.

An a priori power analysis was conducted using G*Power (version 3.1.9.7) to determine the required sample size. Considering that simple mediation models can be statistically approximated using multiple regression analyses, the required sample size was estimated for a linear multiple regression model with two predictors. Assuming a medium effect size (*f*^2^ = 0.15), α = 0.05, and power (1−β) = 0.80, the required sample size was *N* = 68. Our sample (*N* = 104) exceeded this requirement.

All analyses were carried out using Python 3.10, employing the Pandas (McKinney, 2010), NumPy (Harris et al., 2020), and SciPy.stats (Virtanen et al., 2020) libraries.

### 3.1 Mediation analysis

To examine the mediating role of perceived stress in the association between perceived vulnerability and interoception, a rank-based nonparametric mediation approach was implemented. Given violations of normality assumptions, monotonic associations among variables were estimated using Spearman’s rank-order correlation coefficients (Conover, 1999; Field, 2024).

The indirect effect was operationalized using a product-of-coefficients framework (a × b), consistent with contemporary mediation methodology (MacKinnon and Luecken, 2008). Path a (X→M) and path b (M→Y) were estimated using Spearman correlations, and their product provided the point estimate of the indirect effect.

Inference regarding the indirect effect was conducted using nonparametric percentile bootstrapping with 5,000 resamples. For each resample, rank-based path coefficients were recomputed and multiplied to generate an empirical sampling distribution of the indirect effect. Bias-corrected 95% confidence intervals were derived from this distribution. Statistical significance was determined when the confidence interval did not include zero, in line with recommendations for mediation inference based on resampling procedures (Preacher and Hayes, 2008; Tibshirani and Efron, 1993).

The direct effect (*c*′) was estimated using ordinary least squares regression applied to ranked variables to preserve consistency with the rank-based mediation framework (Conover and Iman, 1981). This approach does not rely on normal-theory assumptions and provides robust estimation of indirect effects under conditions of non-normality, skewness, or potential outliers (Wilcox, 2012).

## 4 Results

Here we report descriptive statistics and bivariate associations among the study variables (see Table 1), followed by the results of the mediation analysis testing the indirect effect of perceived stress on the relationship between perceived vulnerability and interoception.

**TABLE 1:** Descriptive statistics and spearman’s rank-order correlations.

Variables	Mean (SD)	Min–max	1	2	3	4
Age	36 (5.2)	30–45	–	−0.232*	–	
Years of education	14.7 (3.43)	4–23				
1. PSS	23.1 (6.48)	6–38				
2. MAIA	3.09 (0.56)	1.62–4.37	−0.295**			
3. PV	5.17 (1.6)	1.25–8.25	0.230*			
4. MP	8.14 (4.13)	0–8	0.126	0..034	0.495***	

Spearman correlation coefficients are presented with significance levels indicated as follows: *p* < 0.05 (*), *p* < 0.01 (**), and *p* < 0.001 (***). PSS, perceived stress; PV, perceived vulnerability; MP, multidimensional poverty (for more details about correlations with MAIA subscales, see Supplementary Table 3). Further information on MAIA subscales is presented in Supplementary Tables 1, 2.

### 4.1 Correlation analysis

A summary of the correlations can be seen in Table 1.

#### 4.1.1 Perceived stress—interoception

The results of the Spearman correlation analysis between perceived stress (PSS) and interoception (MAIA) indicate a significant negative correlation with the subscales “Attention Regulation” (rho = −0.219; *p* = 0.025), “Self-Regulation” (rho = −0.273; *p* = 0.005), “Trusting” (rho = −0.284; *p* = 0.004), and with the total score of the instrument “Total MAIA” (rho = −0.295; *p* = 0.002). These correlations suggest that higher levels of perceived stress are associated with lower interoceptive awareness, as measured by the MAIA.

#### 4.1.2 Perceived stress—perceived vulnerability (subjective) y multidimensional poverty (objective)

In parallel, the correlations between perceived stress and socioeconomic status, both perceived and objective, revealed a significant relationship between PSS and the subjective measure (“PV”) (rho = 0.230; *p* = 0.019). This is particularly relevant, as “PV” not only correlates with perceived stress but also shows a strong relationship with the “Multidimensional Poverty” dimension (rho = 0.495; *p* < 0.001), and when both variables are combined, they correlate significantly with perceived stress (rho = 0.235; *p* = 0.016).

#### 4.1.3 Perceived vulnerability, multidimensional poverty—interoception

Finally, regarding the relationship between perceived and objective socioeconomic status and interoception, significant negative correlations were observed for the following relationships: between the subscales “PV” and “Noticing” (rho = −0.253; *p* = 0.01), “PV” and “Attention Regulation” (rho = −0.277; *p* = 0.004), “PV” and “Emotional Awareness” (rho = −0.213; *p* = 0.03), and “PV” and Total MAIA (rho = −0.232; *p* = 0.018). In contrast, the objective measure of SES did not correlate with any of the interoception subscales.

### 4.2 Mediation analysis

**FIGURE 1:**
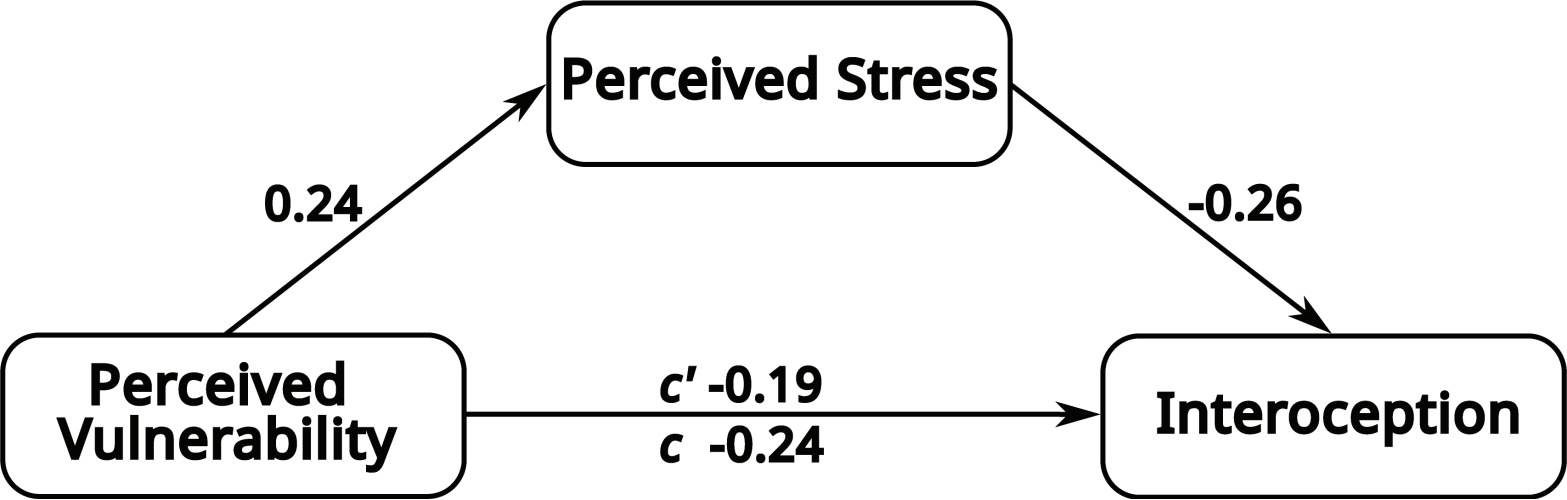
Mediation model examining the role of perceived stress in the association between perceived vulnerability (PV) and interoception. The total effect of PV on interoception was significant (*c* = –0.24, *p* = 0.012) and decreased when accounting for perceived stress (*c*’ = –0.19, *p* = 0.048), indicating partial mediation. All reported values correspond to standardized coefficients. The path *c* represents the total effect of PV on interoception, whereas *c’* represents the direct effect of PV on interoception after controlling for the mediator. See Supplementary Table 3 for additional details.

As shown in Figure 1, the results indicated that higher levels of perceived deprivation (higher PV scores) were associated with higher levels of perceived stress (rho = 0.24, *p* = 0.015), and that higher stress was related to lower interoception (rho = –0.26, *p* = 0.008). The total relationship between perceived vulnerability and interoception was significant (rho = –0.24, *p* = 0.012) and persisted as a weaker but still significant direct effect when controlling for stress (β = –0.19, *p* = 0.048). The indirect effect was significant (a × b = –0.058; 95% CI: –0.15 to –0.005; *p* = 0.022), suggesting partial mediation of approximately 24%. These findings support the hypothesis that perceived stress is a psychological mechanism through which perceived vulnerability impacts interoceptive awareness (for more information, see Supplementary Table 3).

## 5 Discussion

This study examined the relationships among stress, perceived socioeconomic vulnerability (PV), multidimensional poverty (MP), and interoception using a mediation model. Initial correlational analyses showed that perceived stress was associated with both higher PV and greater MP. However, only the subjective measure (i.e., PV) was related to interoception, as evidenced by lower scores on the total MAIA scale and the subdimensions of Noticing, Attention Regulation, and Emotional Awareness. The mediation model further indicated that the association between PV and interoception was partially explained by perceived stress. Together, these findings suggest that conscious interoception is more closely shaped by subjective socioeconomic vulnerability and its psychosocial correlates than by structural indicators of poverty. This represents the first effort to examine the differential effects of perceived vs. objective measures of socioeconomic conditions on interoception.

It is well established that differences in socioeconomic status significantly influence the way individuals perceive and interpret bodily signals (Chentsova-Dutton and Dzokoto, 2014; Freedman et al., 2021; Leão et al., 2025; Ma-Kellams, 2014). Interoceptive sensitivity—such as heartbeat detection accuracy—tends to be lower among individuals or populations exposed to disadvantage or chronic stress (Chentsova-Dutton and Dzokoto, 2014). These effects appear to arise not only from material deprivation but also from exposure multidimensional factors to early adversity, food insecurity, insufficient healthcare access, and other contextual stressors that impact interoceptive processes (Barradas et al., 2021; Feldman et al., 2023; Leão et al., 2025). Our findings partially align with these studies but add an important nuance that has been largely overlooked: the role of the subjective perception of socioeconomic vulnerability in the disruption of interoceptive awareness.

Research has consistently shown that subjective socioeconomic assessments robustly predict physical, emotional, and cognitive outcomes, often more strongly than objective indicators (Demakakos et al., 2008; Hooker et al., 2017; Roy et al., 2019; Shaked et al., 2016; Steen et al., 2020; Navarro-Carrillo et al., 2020; Präg et al., 2016; Quon and McGrath, 2014; Tan et al., 2020; Zhao et al., 2023). Their predictive advantage likely arises because subjective measures capture psychosocial dimensions that traditional indices do not fully reflect, including cumulative adversity, expectations about future stability, perceived family resource constraints, and affective states such as shame or inferiority (Adler et al., 2000; Bosma et al., 2015; Hoebel and Lampert, 2020). Within this framework, our results suggest that these psychosocial components are particularly relevant for understanding why PV—but not multidimensional poverty—was associated with reduced interoceptive awareness. This pattern indicates that individuals’ interpretations and appraisals of their socioeconomic standing may exert a more proximal influence on interoceptive processes than objective socioeconomic indicators alone.

A large body of research indicates that the negative outcomes of subjective socioeconomic vulnerability are attributable to heightened stress and decreased coping abilities (Archibald and Neupert, 2022; Gruenewald et al., 2006; Hoebel and Lampert, 2020; Jackson et al., 2011). Individuals who perceive themselves as disadvantaged tend to show greater threat sensitivity, stronger negative affect and stress responses (Derry et al., 2013; Gruenewald et al., 2006; Rahal et al., 2020). These psychosocial patterns are accompanied by feelings of inferiority, shame, and incompetence that exert disproportionately negative effects on health (Hoebel and Lampert, 2020; Kraft and Kraft, 2023) including the well-known generalized toxic effects of chronic HPA-axis activation (Hooker et al., 2017; Nobles et al., 2013; Steen et al., 2020). At the neurobiological level, perceived socioeconomic vulnerability has been associated with alterations in key limbic regions implicated in stress regulation, such as the anterior cingulate cortex, hippocampus, and amygdala, as well as broader neurovascular and functional vulnerabilities (Mcewen and Gianaros, 2010; Gianaros et al., 2007, 2008; Yang et al., 2016; Yong et al., 2021). Interestingly, such stress-vulnerable areas, particularly insular and ACC have been linked to interoceptive processes (Berntson and Khalsa, 2021; Feldman et al., 2024), representing a common neuroanatomical substrate linking subjective social status to interoception. Taken together, current literature suggests that stress constitutes a compelling mechanism through which PV may disrupt interoceptive processes.

In line with the above, our results show that perceived stress partially mediates the association between perceived vulnerability (PV) and interoceptive awareness. Higher PV was associated with higher stress, which in turn was linked to lower interoception. This pattern confirms that beyond structural disadvantage, perceived social standing carries psychosocial burdens that increase stress vulnerability and reduce coping capacity, ultimately affecting interoception. From a neurophysiological standpoint, these patterns align with integrative models (Alvarez et al., 2022; Lucente and Guidi, 2023; Migeot and Ibáñez, 2025) describing how socioeconomic driven stress disrupts the brain’s allostatic–interoceptive network (AIN), a large-scale system encompassing key nodes of the salience and default mode networks—including the insula, anterior cingulate cortex, amygdala, and hippocampus—responsible for integrating top-down interoceptive predictions with bottom-up viscerosensory input (Kleckner et al., 2017). Chronic stress induces multisystemic load—reflected in inflammatory, metabolic, and cardiovascular markers—that alters the structure and function of key AIN regions, disrupts predictive integration of visceral information, and produces atypical electroencephalographical interoceptive responses (Birba et al., 2022, p. 20; Fava et al., 2019; Franco-O’Byrne et al., 2024, 2025; Hazelton et al., 2025). By incorporating PV and conscious interoceptive processes, our findings refine this neurophysiological literature and highlight a plausible psychological route through which vulnerability becomes biologically embedded.

The partial mediation also indicates that PV contributes to lower interoceptive awareness beyond stress. This effect may reflect cognitive and attentional mechanisms: by involving a representation of scarcity, insecurity, and lack of control over the environment, PV could direct attention toward external threats— such as concerns about stability, social comparisons, or fear of resource loss—thereby reducing attention to internal signals (Leão et al., 2025; Murphy et al., 2018). Altogether, these findings indicate that PV may be associated with lower interoceptive awareness through multiple mechanisms, independent of stress. However, these potential explanations will need to be empirically tested in future studies.

While illuminating potential psychosocial and neurophysiological mechanisms underlying interoception, this study has limitations. Because it is cross-sectional, the mediation should not be interpreted causally. Our approach relied on Spearman correlations to accommodate non-normality and potential non-linearities, with bootstrap-based CIs providing robust estimates. However, we did not control for potential confounders such as sex, which may influence both stress and interoceptive awareness. Future studies should incorporate longitudinal designs and multivariable models to clarify temporal directionality and evaluate alternative computational or modeling frameworks.

## Data Availability

The original contributions presented in this study are included in the article/Supplementary material, further inquiries can be directed to the corresponding author.
