# Supplementary material for: Perceived socioeconomic vulnerability, but not objective poverty, is linked to interoception through perceived stress
Source: Front Psychol. 2026 Apr 1;17:1713385. doi: 10.3389/fpsyg.2026.1713385 (PMC13079641; doi:10.3389/fpsyg.2026.1713385)
Supplement: Supplementary file 1 [file Table_1.DOCX]

**Supplementary tables**

**Table 1**

*Descriptive analysis for the variables of interest*

| **MAIA Subscales** | | **N** | **Mean (SD)** |  |
| --- | --- | --- | --- | --- |
| Notice | | 104 | 3.60 (1.00) |  |
| AR |  | 104 | 3.17 (0.80) |  |
| EA |  | 104 | 3.75 (0.94) |  |
| SR |  | 104 | 3.10 (0.93) |  |
| Trust |  | 104 | 3.51 (1.03) |  |
| Note. Descriptive data for the variables in this study. Abbreviations: “PSS” = Perceived Stress; “MAIA” = Interoception; “PV” = Perceived Vulnerability; “MP” = Multidimensional Poverty. The MAIA subdimensions are abbreviated as follows “Notice” = Noticing; “AR” = Attention Regulation; “EA” = Emotional Awareness; “SR” = Self-Regulation; “Trust” = Trusting. | | | |  |
|  |  |  |  |  |
|  |  |  |  |  |
|  |  |  |  |  |
|  |  |  |  |  |
|  |  |  |  |  |
|  |  |  |  |  |
|  |  |  |  |  |
|  |  |  |  |  |

**Table 2**

|  |  | **PSS** | **PV** | **MP** | **Notice** | **AR** | **EA** | **SR** | **Trust** |  |
| --- | --- | --- | --- | --- | --- | --- | --- | --- | --- | --- |
| **PSS** | Rho |  |  |  |  |  |  |  |  |  |
|  | p value |  |  |  |  |  |  |  |  |  |
| **PV** | Rho | 0.230* |  |  |  |  |  |  |  |  |
|  | p value | 0.019 |  |  |  |  |  |  |  |  |
| **MP** | Rho | 0.126 | 0.495*** |  |  |  |  |  |  |  |
|  | p value | 0.202 | <.001 |  |  |  |  |  |  |  |
| **Notice** | Rho | -0.098 | -0.253** | 0.026 |  |  |  |  |  |  |
|  | p value | 0.324 | 0.01 | 0.795 |  |  |  |  |  |  |
| **AR** | Rho | -0.219* | -0.277** | -0.003 | 0.612*** |  |  |  |  |  |
|  | p value | 0.025 | 0.004 | 0.979 | <.001 |  |  |  |  |  |
| **EA** | Rho | -0.159 | -0.213* | -0.014 | 0.684*** | 0.606*** |  |  |  |  |
|  | p value | 0.107 | 0.03 | 0.887 | <.001 | <.001 |  |  |  |  |
| **SR** | Rho | -0.273** | -0.1 | 0.088 | 0.303** | 0.411*** | 0.544*** |  |  |  |
|  | p value | 0.005 | 0.313 | 0.373 | 0.002 | <.001 | <.001 |  |  |  |
| **Trust** | Rho | -0.284** | -0.174 | -0.06 | 0.314** | 0.416*** | 0.506*** | 0.561*** |  |  |
|  | p value | 0.004 | 0.077 | 0.544 | 0.001 | <.001 | <.001 | <.001 |  |  |
| Note*.* Spearman correlations where * p < .05, ** p < .01, *** p < .001. “PV” = Perceived Vulnerability; “MP” = Multidimensional Poverty. The MAIA subdimensions are abbreviated as follows “Notice” = Noticing; “AR” = Attention Regulation; “EA” = Emotional Awareness; “SR” = Self-Regulation; “Trust” = Trusting. | | | | | | | | | |  |
|  |  |  |  |  |  |  |  |  |  |  |

*Correlation matrix for the studied variables and their subdimensions*

**Table 3**

| **Path** | **Coef.** | ***p* value** |
| --- | --- | --- |
| PV → PSS | 0.2384 | 0.0147 |
| PSS → MAIA | -0.2598 | 0.0077 |
| PV (c)→ MAIA | -0.2447 | 0.0122 |
| PV (c’) → MAIA | -0.1937 | 0.0484 |
| Note*.* The indirect effect (a×b) was estimated using non-parametric bootstrapping (5,000 samples): a×b = –0.058, 95% CI [–0.146, –0.005], p = 0.022. The direct effect of PV on MAIA is represented by *c’*, while the total effect is represented by *c* | | |

*Mediation Analysis*
